# Low ALT, a marker of sarcopenia and frailty, is associated with shortened survival amongst myelodysplastic syndrome patients: A retrospective study

**DOI:** 10.1097/MD.0000000000033659

**Published:** 2023-04-28

**Authors:** Noa Uliel, Gad Segal, Avital Perri, Natia Turpashvili, Reut Kassif Lerner, Edward Itelman

**Affiliations:** a HARVEY Faculty of medicine, Pavia University, Ramat Gan, Israel; b Education Authority, Sheba Medical Center. Affiliated to the Sackler Faculty of Medicine, Tel-Aviv University, Ramat Gan, Israel; c Department of Neurosurgery, Sheba Medical Center. Affiliated to the Sackler Faculty of Medicine, Tel-Aviv University, Ramat Gan, Israel; d Institute of Hematology, Sheba Medical Center. Affiliated to the Sackler Faculty of Medicine, Tel-Aviv University, Ramat Gan, Israel; e Department of Pediatric intensive care, The Edmond and Lily Safra Children’s hospital, Sheba Medical Center, Tel-Hashomer, Israel. Affiliate to Sackler Faculty of Medicine, Tel-Aviv University, Tel-Aviv, Ramat Gan, Israel.

**Keywords:** alanine amino transferase, ALT, frailty, Myelodysplastic syndrome, sarcopenia, survival

## Abstract

Myelodysplastic Syndrome (MDS) is a common blood dyscrasia that mainly affects the elderly population. Several prognostic scores are available utilizing blood count variables and cytogenetic abnormalities, targeting the disease rather than the patient. Sarcopenia and frailty are associated with shortened survival rates in various disease states. Low Alanine Aminotransferase (ALT) levels are a marker of lowered muscle mass and frailty status. This study aimed to examine the correlation between low ALT levels and prognosis in MDS patients. This is a retrospective cohort study. We obtained the demographic, clinical, and laboratory data of patients in a tertiary hospital. Univariate and multivariate models were used to investigate the potential relationship between low ALT level and survival. The final study included 831 patients (median age 74.3 years, Interquartile range 65.6–81.8), and 62% were males. The median ALT level was 15 international units (IU)/L and 233 patients (28%) had low ALT levels (<12 IU/L). Univariate analysis showed that low ALT levels were associated with a 25% increase in mortality (95% confidence interval [CI]: 1.05–1.50, *P* = .014). A multivariate model controlling for age, sex, body mass index, hemoglobin and albumin concentrations, and low ALT levels was still significantly associated with increased mortality (hazard ratio [HR] = 1.25, 95% CI: 1.01–1.56, *P* = .041). Low ALT levels were associated with increased mortality among patients with MDS. Impact: Using ALT as a frailty metric may allow patient-centered, personalized care in this patient population. A low ALT level reflects the pre-morbid robustness of patients and is not intended to replace disease-centered characteristics.

## 1. Introduction

### 1.1. Myelodysplastic syndrome (MDS) patients’ prognosis and treatment personalization

MDS is a group of diverse pathological entities, and several classification systems exist for the staging,^[1]^ prognostication,^[2]^ and treatment personalization of these patients.^[3,4]^ The overall prognosis of patients with MDS is poor, and the application of hypomethylating agents, which potentially prolong patients’ lives, is based on the classification of these patients into low- and high-risk groups.^[5]^ Such patient grouping, namely personalization of disease management, should consider, for example, the patient age, since a poor prognosis of MDS is especially apparent in patients over the age of 60 years.^[6]^ In addition, the patient risk of cardiovascular disease (CVD) and subsequent mortality should be considered, as among low-risk MDS patients, CVD is the second most common cause of death. Alonso-Fernandez-Gatta and co. characterized several biomarkers for increased risk of MDS death from CVD causes,^[7]^ one of which is NT-Pro Brain Natrium uretic peptide peripheral blood concentration. Such biomarkers are investigated and future ones are sought since there is place for improvement of the prognostic evaluation of MDS patients.

MDS is heterogeneous in its clinical manifestations and can affect patient OS, making it difficult to measure its exact outcomes. A prognostic scoring system can help physicians make informed decisions regarding the treatment of patients, and is currently used to evaluate the prognosis of patients with MDS.^[8,9]^ Some of these scoring systems are based on studying each patient cytogenetic determinants, such as the deletion of 5q,^[10]^ while others address mutated oncoproteins.^[11]^ Notwithstanding the above, even after adding the patient disease-centered characteristics to the prognostication process, there is still a need to improve the personalization of the overall management of MDS patients. Starkman et al published a frailty index (FI) for MDS patients. Their FI relies on the accumulated 42-items test results, and although validated, it is cumbersome for routine clinical use.^[12]^ Improving the personalization of prognosis means that the scoring system should incorporate more “patient” characteristics rather than “disease” characteristics. The widely used Revised International Prognostic Scoring System (R-IPSS) is heavily based on disease characteristics, whereas frailty of patients is less considered.^[13]^ We sought to find a new biomarker that would be complementary to the aforementioned biomarker and could be used even before the exact disease and cytological characteristics are evident.

### 1.2. Sarcopenia and frailty as part of personalized medicine of elderly patients

Sarcopenia and frailty assessment are common and consensual aspects of geriatric medicine.^[14–16]^ It could be stated that in this clinical discipline, sarcopenia and frailty are already the face of “personalized medicine,” several steps ahead of other non-geriatric medical domains. In their study of frail, elderly patients, de Vries and co. defined a special physical therapy, intended to reduce frailty, as “patient-centered”—only a step from defining this intervention as personalized medicine, as it should be described.^[17]^ Studies have already shown that sarcopenia and frailty are widespread in the elderly population, approximately one-tenth of community-dwelling older individuals^[18]^ and over 40% on admission to geriatric rehabilitation, which has a potential negative impact on rehabilitation outcomes.^[19,20]^ Many studies have detailed the association between sarcopenia and increased risk of all-cause mortality^[21]^ and many have dealt with the impact of sarcopenia and frailty on patients suffering from diseases that are typically related to aging. Examples of such associations can be found in patients with Parkinson disease (PD). In patients with PD, sarcopenia and frailty were found to be highly prevalent and associated with a higher disease burden.^[22]^ Arterial hypertension is a common chronic disease in the elderly population. In patients with hypertension, frailty has been found to be associated with an increased prevalence of end-organ damage.^[23]^ Sarcopenia has also been associated with a higher risk of nonalcoholic liver disease.^[24]^ Sarcopenia and frailty are very common in patients with devastating Alzheimer dementia, and geriatric interventions are recommended to address these diagnoses in patients with Alzheimer disease patients.^[25]^

### 1.3. Alanine aminotransferase (ALT) as a marker for sarcopenia

ALT is an intracellular transaminase enzyme that catalyzes the conversion of α-keto acids into amino acids via the transfer of amino groups. Hence, it plays a crucial role in essential metabolic pathways such as glycolysis and gluconeogenesis, mainly in the liver and skeletal muscles.^[26]^ Low ALT was previously suggested as a biomarker for sarcopenia, clinically manifested as frailty, and subsequent increased risk of hospitalization and mortality. When diverse patient populations were compared in the same age group,^[27–32]^ the general population of hospitalized patients, elderly individuals prior to rehabilitation due to stroke, heart failure patients in general, and those directed to cardiac rehabilitation, ischemic heart disease patients, patients suffering from atrial fibrillation, and other patient groups. Low ALT was not only found to be associated with dismal outcomes; it was also demonstrated to have a statistically significant correlation with other measures of sarcopenia and frailty, such as the FRAIL score and the L3SMI (striated muscle index at the level of the 3^rd^ lumbar vertebra) score^[33,34]^ derived from analyzing tomographic imaging of patients. Low ALT and frailty predict reduced survival in patients with solid malignancies and poorer patient outcomes, either candidates for surgery or conservative approaches. Previous studies have shown that comorbidities and frailty can predict outcomes in patients with MDS. However, no previous study has addressed the potential association between low ALT levels among patients with MDS and potentially shortened survival.

In this study, we aimed to investigate, through a retrospective analysis of patients’ electronic medical records, the association between low ALT levels and increased long-term mortality in MDS patients. Our goal was to enable a simple and effective tool to be assimilated into future prognostication tools for patients with MDS. We aimed to establish a complementary prognostic tool for the current application of R-IPSS and MDS-FI, as described previously.

## 2. Patients and methods

### 2.1. Patients’ characteristics and flow

**Figure 1.**
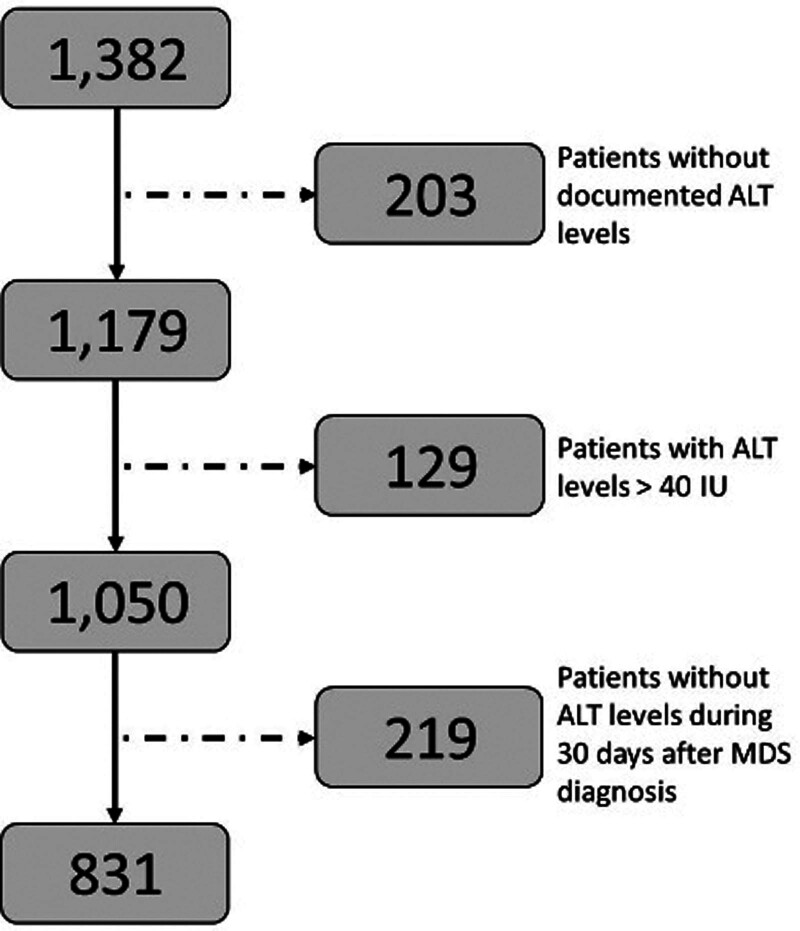
Consort flow diagram of patients.

The patient population in the current study included patients with MDS who were treated (either as outpatients or hospitalized) at the Chaim Sheba Medical Center, the largest tertiary medical center in Israel. Part of them were newly diagnosed in our center and some were referred from smaller, peripheral hospitals. All patient characteristics were obtained from the electronic medical records. These records are routinely used for patient diagnosis and therapy, and are therefore considered reliable and appropriate for patient data retrieval. Prior to patient data collection, the study was approved by the institutional review board (#SMC 9806-22). A total of 1382 MDS patient records were identified, of which only 1179 MDS patients had recorded ALT values during the time they were first admitted. Among these patients, only 1050 had ALT levels below the standard upper limit of 40 international units (IU). We excluded patients with ALT levels higher than 40 IU because these levels are primarily associated with ALT originating from damaged liver tissue (various types of acute and chronic hepatitis) rather than serving as a reliable marker for striated muscle mass. Only 831 patients with MDS had ALT levels within the normal range, 30 days after their primary MDS diagnosis was established. Notably, ALT values were available for sarcopenia and frailty assessments prior to the availability of disease-centered characteristics required for R-IPSS staging. Figure 1 shows the patient consort flow and exclusion diagrams. The final data analysis was performed on 831 patients with MDS whose complete data were available. The patients’ baseline demographic (age and gender) and clinical data (ALT blood activity level, body mass index; body-mass index, background diseases: chronic obstructive pulmonary disease; Chronic obstructive pulmonary disease and congestive heart failure; Congestive heart failure, laboratory parameters: Albumin, Hemoglobin, Platelets and Estimated glomerular filtration rate) were retrieved from the electronic medical records (EMR). The primary outcome was all-cause mortality. Survival data were available for all participants in the Israeli Population National Registry.

### 2.2. Statistical analysis

Continuous variables were expressed as mean ± standard deviation if normally distributed or median with interquartile range if skewed. Normality was determined using the Anderson-Darling and Shapiro–Wilk tests. Categorical variables are presented as frequencies (%). Continuous data were compared using the Student *t* test, variables that were non-normally distributed were tested using the Kruskal–Wallis test and categorical data were compared using the chi-square or Fisher exact test. The log-rank test was used to analyze survival, which was depicted using Kaplan–Meier curves. Univariate Cox regression modeling was used to determine the unadjusted hazard ratio (HR) for the primary outcome and a multivariate model was constructed to examine the correlation and control for possible confounders. An association was considered statistically significant for a 2-sided *P* value of <.05. All analyses were performed using the R software version 4.1.0 (R Foundation for Statistical Computing).

## 3. Results

The final study population included 831 patients with a median age of 74.3 years (interquartile range 65.6–81.8), and 62% were males. The mean time for follow-up was 29.4 months (medical 14.4 months). The median ALT levels in the study population were 15 IU/L, and 233 patients (28%) had low ALT levels, defined as levels lower than 12 IU/L. Males comprised only 55% of patients with low ALT levels, while their proportion was higher (64%) among patients with ALT values >12IU/L (*P* = .025). Patients with low ALT values also had lower albumin values 3.6 (3.2–3.9) versus 3.7 (3.3–4), (*P* value = .008) and lower hemoglobin values 9.15 (8.16–10.23) versus 9.47 (8.45–10.66), (*P* value = .003). Other patient demographics and characteristics were detailed according to ALT levels (lower or ≥ 12 IU/L) (Table 1).

**Table 1:** Patients’ characteristics according to ALT level.

Patient characteristic	ALT < 12 IU/L	ALT ≥ 12 IU/L	*P* value
N = 831	N = 223 (28%)	N = 608 (72%)	
ALT (IU/L), median (IQR)	9 (7–10)	18 (14–24)	<.001
Age (yr), median (IQR)	74.7 (65.5–83.5)	74 (65.5–81.1)	.258
Male, N (%)	123 (55)	389 (64)	.025
BMI, median (IQR)	24.65 (22.3–27.9)	26.1 (23.2–29.1)	.012
COPD, N (%)	10 (4.5)	26 (4.3)	1
CHF, N (%)	19 (8.5)	50 (8.2)	1
Albumin (g/dL), median (IQR)	3.6 (3.2–3.9)	3.7 (3.3–4)	.008
Hb (g/dL), median (IQR)	9.15 (8.16–10.23)	9.47 (8.45–10.66)	.003
PLT (10^9/L), median (IQR)	100 (44–183)	106 (45–183)	.816
EGFR (mL/min), median (IQR)	66.2 (43.7–86.6)	68.7 (49.4–88.2)	.31

ALT = alanine aminotransferase, BMI = body mass index, CHF = congestive heart failure, COPD = chronic obstructive pulmonary disease, EGFR = estimated glomerular filtration rate, Hb = hemoglobin, IU = international units, IQR = interquartile range, PLT = platelet.

### 3.1. Univariate analysis

**Figure 2.**
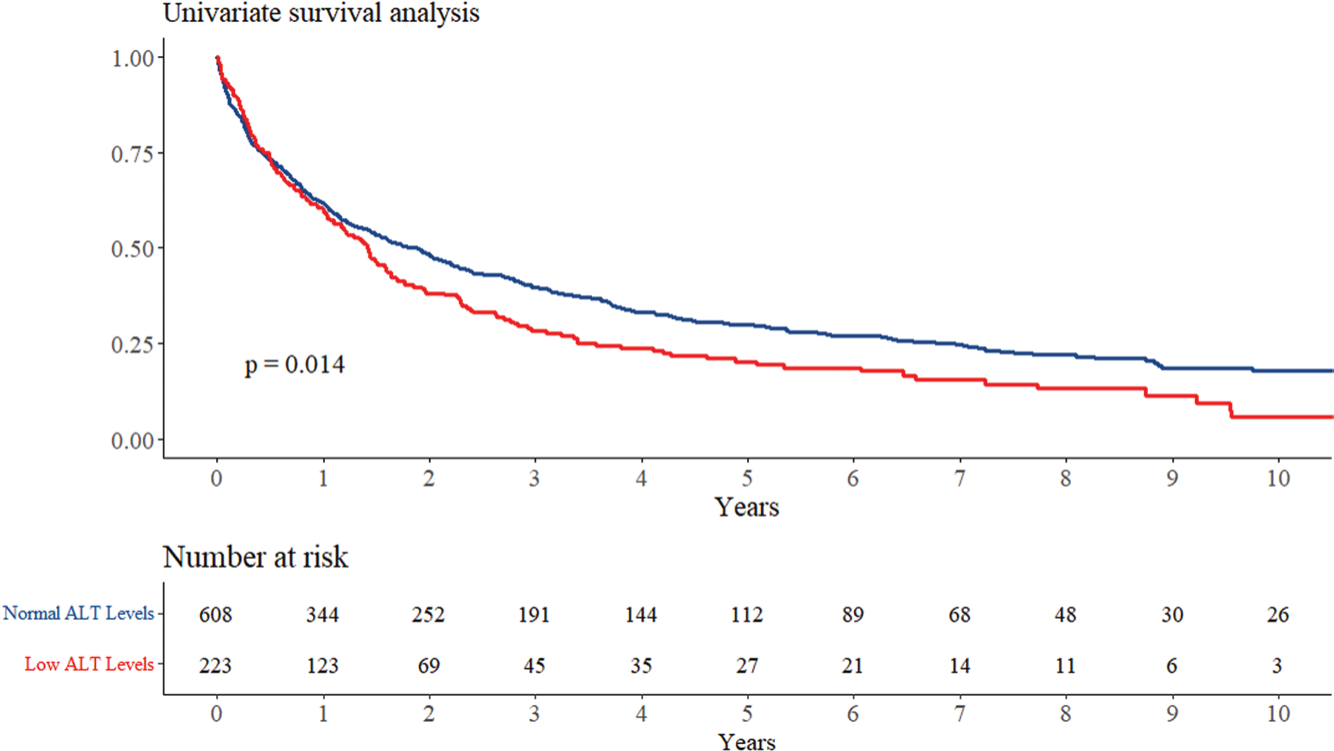
Kaplan Meir survival analysis according to ALT levels. ALT = alanine aminotransferase.

Univariate analysis showed that low ALT levels were associated with a significant 25% increase in mortality (95% confidence interval [CI]: 1.05–1.50, *P* = .014). Figure 2 shows the Kaplan–Meier curve for crude survival analysis according to ALT levels.

### 3.2. Multivariate analysis

In a multivariate model (Table 2) we opted to use variables that were either significantly different among the population or of known prognostic implications among patients with MDS. The analysis was controlled for age, sex, body mass index, hemoglobin level, white blood cell count, platelets, and albumin concentration, low ALT levels were still associated with increased mortality (HR = 1.27, 95% CI: 1.02–1.58, *P* = .030).

**Table 2:** Multivariate analysis.

Multivariate analysis		
	Hazard ratio	*P*
ALT < 12 IU/L	1.27 [1.02, 1.58]	.030
Male gender	1.20 [0.98, 1.48]	.081
Age	1.03 [1.02, 1.04]	<.001
Body mass index	1.00 [0.98, 1.02]	.873
Hemoglobin ≤ 14 gr/dL	1.45 [0.64, 3.26]	.369
Albumin < 3 gr/dL	1.87 [1.38, 2.51]	<.001
White blood cells/mcl	1.02 [1.01, 1.02]	<.001
Platelets/mcl	1.00 [1.00, 1.00]	<.001

ALT = alanine aminotransferase, IU = international units.

## 4. Discussion

While reviewing the current literature for evidence supporting personalized medicine for MDS patients, the terms “personalized” and “precise” are frequently used interchangeably. For example, in their review of “personalized medicine for TP53 mutated MDS and acute myeloid leukemia,^[35]^ the authors named the precision and targeted therapy for TP53 as “personalized.” Indeed, the quest for better suitability of therapy will lead to better disease targeting. Nevertheless, their findings do not align with personalized therapy for patients. Instead, they have improved the precision of targeted therapy. Although the fact that their better targeted therapy will increase treatment efficacy, it will not necessarily be well tolerated by frail patients with MDS. In her recent review “Advances in myelodysplastic syndrome,” Valeria Santini screened recent advancements in targeting molecular biomarkers of this disease.^[36]^ It seems that alongside such research advancements, little is known about personalizing treatment according to the frailty/robustness potential of the patient. Disease-centered classification systems, such as R-IPSS,^[13]^ include cytogenetic parameters that are unknown upon patient admission and are less concentrated on the patient baseline robustness. Other frailty-centered evaluation tools, such as the MDS-IF scale,^[12]^ are cumbersome and less attractive for real-time evaluation of newly diagnosed MDS patients.

**Figure 3.**
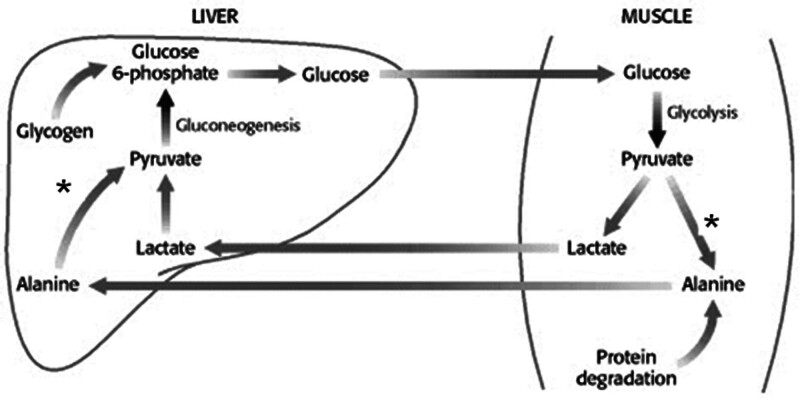
ALT (SGPT) activity (asterisk) in the liver and skeletal muscle catalyzes the bidirectional transformation of Alanine and Pyruvate. ALT = alanine aminotransferase, SGPT = serum glutamic pyruvic transaminase.

Frailty, as the phenotypic and functional translation of sarcopenia, significantly affects patients’ potential to fight back disease, both acute and chronic. Starting from Morley classic descriptions of sarcopenia and frailty,^[37]^ sarcopenia and frailty assessment into the personalization of medical therapy is well established through the consensus statements of the European innovation partnership on active and healthy aging.^[38]^ Our group, as well as others, have previously established an association between low peripheral blood levels of ALT and lower mass of skeletal striated muscle. ALT, also known as serum glutamic pyruvic transaminase, is a fundamental enzyme that plays a key role in the intermediary metabolism of glucose and amino acids^[39,40]^ (Fig. 3).

ALT catalyzes a bidirectional molecular process in various tissues, including skeletal muscles and the liver. As ALT activity in the liver is approximately 3000 times higher than that in the serum, its main purpose in clinical settings is to rule out—and assess, as in the case of hepatitis—hepatocellular injury from various causes. The amount of ALT in tissues other than the liver, such as the skeletal muscle tissue, is much lower.^[40]^ The catalytic activity of ALT is facilitated by *P*-5-P, Pyridoxal 5 phosphate, a metabolic derivative of vitamin B6, which acts as a co-factor for this enzyme. The upper limit of normal for ALT peripheral blood activity was approximately 40 IU. Above this level of activity in the blood, it is assumed that ALT is released from cellular tissues (mainly liver hepatocytes); therefore, ALT levels > 40 IU should not be interpreted in relation to muscle mass, sarcopenia, and frailty. Consequently, patients with ALT measurements should be evaluated using methods other than ALT levels and were excluded from this study.

After preliminary publications associated low ALT levels with lower skeletal muscle mass and increased long-term mortality in the older population,^[40,41]^ several publications have described a more comprehensive association between decreased levels of ALT activity in the peripheral blood, sarcopenia, frailty, and an increased risk of all-cause mortality in middle-aged, heterogeneous populations. In addition, associations were found between low ALT levels as a marker for the above parameters and a decreased potential for patients’ rehabilitation processes.

Le Couteur and co. investigated the possible relationships between blood tests for liver function and injury (such as ALT) on the 1 hand, age, frailty, and survival.^[42]^ This study included 1673 community-dwelling men aged ≥70 years. They found that ALT blood activity was lower in older participants. Participants with ALT below the median at baseline had reduced survival (HR 2.10, 95% CI:1.53–2.87) by up to 4.9 years. In addition, they found that low ALT levels were associated with frailty (odds ratio 3.54, 95% CI: 2.45–5.11), and the relationship between ALT levels and survival disappeared once frailty and age were included in the survival analysis. Their findings reinforced the idea that low ALT activity is a predictor of reduced survival, associated with frailty and increasing age. In a retrospective cohort study, Gringauz and co. studied the association between low ALT levels and poor rehabilitation outcomes after the operative repair of hip fractures.^[20]^ In a retrospective analysis of 490 elderly patients (over 60 years, mean 82.9 ± 6.7 years) admitted to rehabilitation following hip fracture surgery, of whom 82% were women, rehabilitation outcomes were assessed by Functional Independence Measure (FIM) scores. Patients with ALT blood levels of > 40 IU/l were excluded from the study. The cohort was divided into 2 groups: patients with ALT of more than 10 IU/l and patients with ALT of less than or equal to 10 IU/l. Upon rehabilitation discharge, the FIM outcome measures (motor, cognitive, gain, and efficiency) were significantly higher in patients with ALT levels > 10 IU/l relative than in patients with ALT levels ≤ 10 IU/l (*P* < .05). Logistic regression analysis adjusted for age and sex showed that patients with ALT levels > 10 IU/l were more likely to have higher total FIM scores, cognitive FIM scores, and FIM efficiency when discharged. The authors concluded that high-normal ALT blood levels prior to rehabilitation were associated with better outcomes in older adults after hip fracture surgery. Peltz-Sinvani and co. performed a retrospective analysis of the BIP (Bezafibrate Infarction Prevention) study with regard to the association between low ALT levels and long-term mortality.^[27]^ The analysis included 6575 patients without known liver pathology, who were followed up for a median period of 22.8 years. The cumulative probability of all-cause mortality was significantly higher in the low-ALT group than patients the higher ALT levels (65.6% vs 58.4%; log-rank *P* < .001). Multivariate analysis adjusted for multiple other well-established predictors of mortality showed that low ALT level was independently associated with an 11% greater long-term mortality risk (HR: 1.11, 95% CI: 1.03–1.19; adjusted *P* < .01]). The authors concluded that low ALT levels were associated with increased long-term mortality among middle-aged patients with stable coronary heart disease. Ramati and co. conducted a large-scale retrospective survey of 96,726 patients hospitalized in various departments of a tertiary general hospital.^[43]^ They found that low ALT levels were common in this large, heterogeneous population; over 1-third of hospitalized patients had low ALT activity in their blood samples.

The above findings of previous studies provide significant support for the need to assimilate ALT level examination as part of each elderly population of either the diseased or healthy population. Patients with MDS were not excluded from the study. The usability of ALT measurements in the population of patients with MDS should be considered because most have not presented the exclusion criteria for using ALT as a biomarker for sarcopenia (e.g., advanced kidney failure, active or chronic hepatitis, and dependence on the regular usage of anti-PD medications). In most cases, disease in the elderly makes MDS highly suitable for implementing scoring systems intended to diagnose frailty and sarcopenia. Unfortunately, elderly MDS patients are no less elderly than the general MDS patient population; therefore, they are eligible for frailty diagnosis based on sequential ALT measurements, among other parameters.

## 5. Conclusions

After exclusion of a minor portion of MDS patients (those with signs of biochemical hepatitis, advanced renal insufficiency, and use of dopaminergic and anti-Parkinson medications), low ALT values measured during the initial MDS diagnosis were found to be associated with shortened survival of MDS patients. This finding was demonstrated both in the crude survival analysis and in univariate and multivariate analyses. The high availability of ALT measurements makes them a good tool for prognostication and a good candidate for future assimilation into scoring systems aimed at personalizing MDS patient therapy. It must be stated that low ALT reflects the pre-morbid robustness/frailty status of patients rather than their disease characteristics, which should be included in the overall assessment of patients with MDS.

### 5.1. Limitations

This was a single-center retrospective study. Therefore, the generalization of these findings should be sought. In addition, ALT measurements should be implemented in larger and more diverse MDS populations before assimilation into the international prognostic standards and tools. Our study focused on the personalization of prognosis rather than on precise medicine. Therefore, we did not include IPSS cytogenetics in our analysis. Prognostication methods that consider these factors are important and will continue to be the cornerstone in the treatment of patients with MDS.

## Author contributions

**Conceptualization:** N. Uliel, Gad Segal, Natia Turpashvili.

**Data curation:** N. Uliel, Gad Segal, Natia Turpashvili, Edward Itelman.

**Formal analysis:** N. Uliel, Gad Segal, Edward Itelman.

**Methodology:** N. Uliel, Gad Segal, Avital Perri, Natia Turpashvili, Edward Itelman.

**Software:** Edward Itelman.

**Supervision:** Gad Segal, Avital Perri, Reut Kasif-Lerner.

**Validation:** Reut Kasif-Lerner, Edward Itelman.

**Visualization:** Reut Kasif-Lerner.

**Writing – original draft:** N. Uliel, Gad Segal, Avital Perri, Natia Turpashvili, Reut Kasif-Lerner, Edward Itelman.

**Writing – review & editing:** N. Uliel, Gad Segal, Avital Perri, Natia Turpashvili, Reut Kasif-Lerner.

## References

[1] Zeidan AM Shallis RM Wang R. Epidemiology of myelodysplastic syndromes: why characterizing the beast is a prerequisite to taming it. Blood Rev. 2019;34:1–15. 30314642 10.1016/j.blre.2018.09.001

[2] Tsuruda K Hasegawa H Fuchigami M. [Classification and clinical findings of myelodysplastic syndromes]. Rinsho Byori. 2014;62:359–68. 25022065

[3] Santini V Prebet T Fenaux P. Minimizing risk of hypomethylating agent failure in patients with higher-risk MDS and practical management recommendations. Leuk Res. 2014;38:1381–91. 25444075 10.1016/j.leukres.2014.09.008

[4] Bouchla A Thomopoulos TP Papageorgiou SG. Predicting outcome in higher-risk myelodysplastic syndrome patients treated with azacitidine. Epigenomics. 2021;13:1129–43. 34291653 10.2217/epi-2021-0124

[5] Kataria A Jaegerskog E Jindal R. MDS-447 a systematic literature review of the economic burden in patients with myelodysplastic syndromes. Clin Lymphoma Myeloma Leuk. 2022;22(Suppl 2):S315.

[6] Haroun F Solola SA Nassereddine S. PD-1 signaling and inhibition in AML and MDS. Ann Hematol. 2017;96:1441–8. 28643044 10.1007/s00277-017-3051-5

[7] Alonso-Fernandez-Gatta M Martin-Garcia A Martin-Garcia AC. Predictors of cardiovascular events and all-cause of death in patients with transfusion-dependent myelodysplastic syndrome. Br J Haematol. 2021;195:536–41. 34180544 10.1111/bjh.17652

[8] Nazha A. The MDS genomics-prognosis symbiosis. Hematology Am Soc Hematol Educ Program. 2018;2018:270–6. 30504321 10.1182/asheducation-2018.1.270 PMC6246025

[9] Scalzulli E Pepe S Colafigli G. Therapeutic strategies in low and high-risk MDS: what does the future have to offer? Blood Rev. 2021;45:100689. 32253020 10.1016/j.blre.2020.100689

[10] Gurnari C Piciocchi A Soddu S. Myelodysplastic syndromes with del(5q): a real-life study of determinants of long-term outcomes and response to lenalidomide. Blood Cancer J. 2022;12:132. 36071048 10.1038/s41408-022-00724-3 PMC9452671

[11] Lieu YK Liu Z Ali AM. SF3B1 mutant-induced missplicing of MAP3K7 causes anemia in myelodysplastic syndromes. Proc Natl Acad Sci U S A. 2022;119:1–11. 10.1073/pnas.2111703119 PMC8740767 34930825

[12] Starkman R Alibhai S Wells RA. An MDS-specific frailty index based on cumulative deficits adds independent prognostic information to clinical prognostic scoring. Leukemia. 2020;34:1394–406. 31811236 10.1038/s41375-019-0666-7

[13] Greenberg PL Tuechler H Schanz J. Revised international prognostic scoring system for myelodysplastic syndromes. Blood. 2012;120:2454. 22740453 10.1182/blood-2012-03-420489 PMC4425443

[14] Landi F Calvani R Tosato M. Age-related variations of muscle mass, strength, and physical performance in community-dwellers: results from the milan EXPO survey. J Am Med Dir Assoc. 2017;18:88.e17–24. 10.1016/j.jamda.2016.10.007 27914849

[15] Marzetti E Calvani R Tosato M. Sarcopenia: an overview. Aging Clin Exp Res. 2017;29:11–7. 28155183 10.1007/s40520-016-0704-5

[16] Beaudart C McCloskey E Bruyère O. Sarcopenia in daily practice: assessment and management. BMC Geriatr. 2016;16:1–10. 27716195 10.1186/s12877-016-0349-4 PMC5052976

[17] de Vries NM Staal JB van der Wees PJ. Patient-centred physical therapy is (cost-) effective in increasing physical activity and reducing frailty in older adults with mobility problems: a randomized controlled trial with 6 months follow-up. J Cachexia Sarcopenia Muscle. 2016;7:422–35. 27239405 10.1002/jcsm.12091 PMC4864107

[18] Sánchez-García S García-Peña C Salvà A. Frailty in community-dwelling older adults: association with adverse outcomes. Clin Interv Aging. 2017;12:1003–11. 28721028 10.2147/CIA.S139860 PMC5498785

[19] Pongpipatpaiboon K Kondo I Onogi K. Preliminary study on prevalence and associated factors with sarcopenia in a geriatric hospitalized rehabilitation setting. J Frailty Aging. 2018;7:47–50. 29412442 10.14283/jfa.2017.40

[20] Gringauz I Weismann J Justo D. Alanine aminotransferase blood levels and rehabilitation outcome in older adults following hip fracture surgery. Int J Rehabil Res. 2018;41:41–6. 29068797 10.1097/MRR.0000000000000258

[21] Liu P Hao Q Hai S. Sarcopenia as a predictor of all-cause mortality among community-dwelling older people: a systematic review and meta-analysis. Maturitas. 2017;103:16–22. 28778327 10.1016/j.maturitas.2017.04.007

[22] Vetrano DL Pisciotta MS Laudisio A. Sarcopenia in Parkinson disease: comparison of different criteria and association with disease severity. J Am Med Dir Assoc. 2018;19:523–7. 29396191 10.1016/j.jamda.2017.12.005

[23] Tabara Y Kohara K Ochi M. Association of office-based frailty score with hypertensive end organ damage in the J-SHIPP cross-sectional study. Int J Cardiol. 2016;216:25–31. 27135153 10.1016/j.ijcard.2016.04.135

[24] Hong HC Hwang SY Choi HY. Relationship between sarcopenia and nonalcoholic fatty liver disease: the Korean sarcopenic obesity study. Hepatology. 2014;59:1772–8. 23996808 10.1002/hep.26716

[25] Hirose D Hanyu H Fukasawa R. Frailty and sarcopenia in subjects with Alzheimer’s disease with or without cerebrovascular disease. Geriatr Gerontol Int. 2016;16:1235–6. 27870509 10.1111/ggi.12709

[26] Cox MM Nelson DL. Lehninger Principles of Biochemistry. Sixth. Springer Nature Switzerland AG. Part of Springer Nature: W.H.Freeman & Co Ltd; 2013.

[27] Peltz-Sinvani N Klempfner R Ramaty E. Low ALT levels independently associated with 22-year all-cause mortality among coronary heart disease patients. J Gen Intern Med. 2016;31:209–14. 26245731 10.1007/s11606-015-3480-6 PMC4720656

[28] Lasman N Shalom M Turpashvili N. Baseline low ALT activity is associated with increased long-term mortality after COPD exacerbations. BMC Pulm Med. 2020;20:1–6. 32393221 10.1186/s12890-020-1169-z PMC7216624

[29] Itelman E Segev A Ahmead L. Low ALT values amongst hospitalized patients are associated with increased risk of hypoglycemia and overall mortality: a retrospective, big-data analysis of 51 831 patients. QJM. 2022;114:843–7. 32642782 10.1093/qjmed/hcaa219

[30] Anani S Goldhaber G Brom A. Frailty and sarcopenia assessment upon hospital admission to internal medicine predicts length of hospital stay and re-admission: a prospective study of 980 patients. J Clin Med. 2020;9:1–12. 10.3390/jcm9082659 PMC7464238 32824484

[31] Segev A Itelman E Avaky C. Low ALT levels associated with poor outcomes in 8700 hospitalized heart failure patients. J Clin Med. 2020;9:1–10. 10.3390/jcm9103185 PMC7600048 33008125

[32] Segev A Itelman E Beigel R. Low ALT levels are associated with poor outcomes in acute coronary syndrome patients in the intensive cardiac care unit. J Cardiol. 2022;79:385–90. 34696927 10.1016/j.jjcc.2021.10.001

[33] Portal D Melamed G Segal G. Sarcopenia as manifested by L3SMI is associated with increased long-term mortality amongst internal medicine patients-a prospective cohort study. J Clin Med. 2022;11:3500. 35743568 10.3390/jcm11123500 PMC9224962

[34] Portal D Hofstetter L Eshed I. L3 skeletal muscle index (L3SMI) is a surrogate marker of sarcopenia and frailty in non-small cell lung cancer patients. Cancer Manag Res. 2019;11:2579–88. 31114324 10.2147/CMAR.S195869 PMC6497853

[35] Cluzeau T Loschi M Fenaux P. Personalized medicine for TP53 mutated myelodysplastic syndromes and acute myeloid leukemia. Int J Mol Sci. 2021;22:1–9. 10.3390/ijms221810105 PMC8471083 34576266

[36] Santini V. Advances in myelodysplastic syndrome. Curr Opin Oncol. 2021;33:681–6. 34474438 10.1097/CCO.0000000000000790

[37] Morley JE. Frailty and sarcopenia in elderly. Wien Klin Wochenschr. 2016;128(Suppl 7):439–45. 27670855 10.1007/s00508-016-1087-5

[38] Longobucco Y Benedetti C Tagliaferri S. Proactive interception and care of frailty and multimorbidity in older persons: the experience of the European innovation partnership on active and healthy ageing and the response of parma local health trust and lab through European projects. Acta Biomed. 2019;90:364–74. 31125023 10.23750/abm.v90i2.8419 PMC6776195

[39] Senior JR. Alanine aminotransferase: a clinical and regulatory tool for detecting liver injury-past, present, and future. Clin Pharmacol Ther. 2012;92:332–9. 22871997 10.1038/clpt.2012.108

[40] Kim WR Flamm SL Di Bisceglie AM. Serum activity of alanine aminotransferase (ALT) as an indicator of health and disease. Hepatology. 2008;47:1363–70. 18366115 10.1002/hep.22109

[41] Elinav E Ackerman Z Maaravi Y. Low alanine aminotransferase activity in older people is associated with greater long-term mortality. J Am Geriatr Soc. 2006;54:1719–24. 17087699 10.1111/j.1532-5415.2006.00921.x

[42] Le Couteur DG Blyth FM Creasey HM. The association of alanine transaminase with aging, frailty, and mortality. J Gerontol A Biol Sci Med Sci. 2010;65:712–7. 20498223 10.1093/gerona/glq082 PMC4085878

[43] Ramaty E Maor E Peltz-Sinvani N. Low ALT blood levels predict long-term all-cause mortality among adults. a historical prospective cohort study. Eur J Intern Med. 2014;25:919–21. 25468741 10.1016/j.ejim.2014.10.019

